# Effect of sulfasalazine on ferroptosis during intestinal injury in rats after liver transplantation

**DOI:** 10.1038/s41598-024-58057-z

**Published:** 2024-03-28

**Authors:** Wei Wu, Wenhao Bu, Yongxing Tan, Yongwang Wang

**Affiliations:** 1 Department of Anesthesiology, CR & WISCO General Hospital, Wuhan University of Science and Technology, Wuhan, 430080 China; 2 Department of Anesthesiology, Maternal and Child Health Hospital of Hubei Province, Tongji Medical College, Huazhong University of Science and Technology, Wuhan, 430070 China; 3 Department of Anesthesiology, Guilin Municipal Hospital of Traditional Chinese Medicine, Guilin, 541000 China; 4 Department of Anesthesiology, Affiliated Hospital of Guilin Medical University, Guilin, 541000 China

**Keywords:** Liver-transplantation, Ferroptosis, Intestine, Sulfasalazine, Cell biology, Drug discovery, Medical research

## Abstract

Using a rat autologous orthotopic liver transplantation (AOLT) model and liver cold ischemia–reperfusion (I/R)-induced intestinal injury, we clarified whether ferroptosis occurred in rat AOLT cold I/R-induced intestinal injury. Additionally, the role and possible mechanism of the ferroptosis activator sulfasalazine (SAS) in intestinal injury-induced ferroptosis in rats with AOLT liver cold I/R were investigated. Sixty specific pathogen free (SPF)-grade adult male Sprague‒Dawley (SD) rats were randomly divided into 5 groups using the random number table method (*n* = 12). Six rats were randomly selected at 6 hour (h) and 24 h after I/R. Inferior vena cava blood specimens were collected from the portal vein (PV) opening at 6 h and 24 h. The concentrations of serum malondialdehyde (MDA), serum interleukin 6 (IL-6) were determined by enzyme-linked immunosorbent assay (ELISA). Ileal tissue was obtained from the PV opening in rats in each group at 6 h and 24 h, and ileal tissue sections were observed under light microscopy. The contents of intestinal MDA, superoxide dismutase (SOD), glutathione(GSH), glutathione peroxidase 4 (GPX4), and tissue iron were determined by ELISA, and the expression of GPX4 and the cysteine glutamate reverse transporter light chain protein (xCT) was determined by Western blot. The experimental results show that ferroptosis is involved in the pathophysiological process of intestinal injury induced by cold hepatic ischemia–reperfusion in AOLT rats. In addition, SAS (500 mg/kg) may inhibit the cystine/glutamate antiporters (System Xc¯)/GSH/GPX4 signal axis in intestinal injury induced by cold I/R in rat AOLT liver, or iron overload after reperfusion, causing a massive accumulation of L-ROS and activating cellular ferroptosis, further aggravate the intestinal injury.

## Introduction

Perioperative liver transplantation (LT) can not only cause liver damage but also cause brain, heart, lung, kidney, and especially intestinal damage^1–3^. Perioperative ischemia–reperfusion (I/R) after LT can lead to oxidative stress and inflammatory responses, which can lead to decreased intestinal motility and impaired intestinal mucosal barrier function^4,5^. Ferroptosis is a newly discovered type of programmed cell death characterized by the excessive accumulation of iron-dependent lipid reactive oxygen species (L-ROS) through processes associated with various metabolic regulatory pathways, such as iron metabolism, lipid metabolism and amino acid metabolism^6,7^. Studies have shown that ferroptosis in intestinal epithelial cells is involved in the pathophysiological mechanism of intestinal I/R injury^8,9^. In addition, some studies have found that SAS not only inhibits the NF-κB pathway, but also inhibits the ferroptosis signal pathway System Xc^—^^10,11^. The aim of this study was to evaluate the role of ferroptosis in perioperative LT intestinal injury in rats and further clarify the possible mechanism of the ferroptosis activator sulfasalazine (SAS) in perioperative LT-induced intestinal injury in rats.

## Materials and methods

### Experimental animal and groups

Sixty healthy adult SPF-grade male Sprague‒Dawley (SD) rats, aged 10–12 weeks and weighing 300–350 g, were provided by Hunan Silaike Jingda Laboratory Animal Co. Ltd. (experimental animal license number: SCXK (Xiang) 2019-0004). All animal experimental procedures and handling were approved by the Experimental Animal Ethics Committee of Guilin Medical University (No: GLMC-IACUC-2021014), all experiments were performed in accordance with relevant regulations and be reported as described by the ARRIVE guidelines. Using the random number table method, the rats were randomly divided into 5 groups (*n* = 12). At 6 h and 24 h after I/R, 6 rats were randomly selected. (1) In the sham-operated group (Sham group), the abdomen was opened, the corresponding ligaments and blood vessels were freed and exposed, the abdominal cavity was washed with warm normal saline, and then the abdomen was closed. (2) In the autologous orthotopic liver transplantation (AOLT) I/R group (I/R group), after the abdomen was opened, the corresponding ligaments and blood vessels were freed and exposed, and the abdominal cavity was washed with warm normal saline, the portal vein (PV), suprahepatic vena cava (SHVC), infrahepatic vena cava (IHVC), and the proper hepatic artery were sequentially blocked. A perfusion device was used to deliver 4 °C sodium lactate Ringer's solution for liver perfusion via the PV. A small portion of the IHVC was cut as the outflow tract. After 30 ± 1 min, the blood flow of the clamped blood vessels was restored, and the liver changed from yellowish brown to bright red. After flushing the abdominal cavity, the abdomen was closed. (3) In the ferrostain-1 group (I/R + Fer-1 group), 5 mg/kg ferrostain-1 (Catalog No.: HY-100579, MCE, China) was injected intraperitoneally 1 h before surgery; other procedures were the same as those in the I/R group. (4) In the sulfasalazine group (I/R + SAS group), 7 days before surgery, 500 mg/kg SAS (Catalog No.: HY-14655, MCE, China) was intraperitoneally injected twice per day; other procedures were the same as those in the I/R group. (5) In the SAS + ferrostatin-1 group (I/R + SAS + Fer-1 group), 7 days before surgery, 500 mg/kg SAS was intraperitoneally injected twice per day at, and 5 mg/kg ferrostatin-1 was intraperitoneally injected 1 h before surgery; other procedures were the same as those in the I/R group.

### Model establishment

This study refers to the literature for the establishment of an I/R-induced intestinal injury model after autologous orthotopic LT in SD rats^12–14^. Before modeling, rats were fasted for 8 h and were allowed to drink water freely. The rats were intraperitoneally injected with 3% pentobarbital sodium (0.2 ml/100 g) to maintain anesthesia, Intraperitoneal injection of 0.005% fentanyl (0.16 ml/100 g) to relieve pain. Each rat was fixed in the supine position, hair was removed from the abdomen to prepare the skin, the skin was disinfected with iodophor, and sterilized surgical towels were laid out. A midline incision was made in the abdomen, followed by layer-by-layer incisions into the abdominal cavity. The hepatic falciform ligament and the left deltoid ligament were dissected, the liver was turned to the left to open the right posterior peritoneum, and the SHVC and IHVC were carefully bluntly separated. The hepatoduodenal ligament was then cut to expose the PV and proper hepatic artery. The proper hepatic artery, PV, and IHVC were sequentially clamped using noninjury vascular clips, and hepatic blood flow was completely blocked, i.e., the anhepatic phase. The liver was perfused with 1 mL of heparinized saline (25 U/mL) to clear the blood in the liver to prevent thrombosis and replenish the blood volume of the rats; then, the SHVC was blocked. A No. 4 infusion needle was used to infuse the liver with 4 °C lactated Ringer's solution through the PV, and a small hole (1–2 mm) was cut at the site above the IHVC block as an outflow tract. The perfusion rate of sodium lactate Ringer's solution was 6–8 mL/min, and the perfusion pressure was 10 kPa. Fine ice chips were used to cover the liver surface, and the cold perfusion time was 30 ± 1 min. The liver gradually changed from bright red to earthy yellowish brown, indicating successful modeling. After perfusion, the PV needle site and the outflow tract on the IHVC were carefully sutured using an 8-0 noninjury suture. The SHVC, IHVC, PV, and proper hepatic arteries were successively opened. After hepatic blood flow was restored, the liver changed from yellowish brown to red. After rinsing the abdominal cavity with warm normal saline, the abdomen was closed.

### Specimen collection

At 6 h and 24 h after the anhepatic phase, the rats were again anesthetized, and 2 ml of inferior vena cava blood was collected and placed in a BD tube. After centrifugation, the specimen was stored in a – 80 °C freezer. After collecting blood, the left ventricle was perfused with 50 ml of 4 °C heparinized saline. The right atrial appendage was used as the outflow tract. Adequate perfusion was performed to remove residual blood from the heart and body, and then, rat ileal tissue was obtained and stored in a – 80 °C freezer.

### Detection indicators

#### Hematoxylin–eosin (H&E) staining and observation of intestinal pathological changes in rats in each group under a light microscope

The ileal tissue of rats was collected and fixed in 4% paraformaldehyde solution for more than 24 h. After embedding the specimens in paraffin, the specimens were sectioned (the thickness of intestinal tissue section is 5 μm) and subjected to H&E staining. The intestine sections were observed under an optical microscope (EVOS M5000, Thermo Fisher Scientific, USA) (200×), and the pathology results were described using Chiu's scoring method. This operation was carried out by two professionals from the Department of Pathology of the affiliated Hospital of Guilin Medical University.

#### Wet/dry weight (W/D) ratio of intestinal tissue

At 6 and 24 h after AOLT liver reperfusion, the ileal tissue of 5 cm in each group was taken and rinsed with 0.9% normal saline, and then weighed as wet weight; Then put it in an oven at 80℃ and weigh it again after baking for 24 h, which is called dry weight. The ratio of intestinal W/D in each experimental group was calculated in turn.

#### Determination of Fe^2+^ content in intestinal tissue

Approximately 0.1 g of intestinal tissue and 1 mL of extract were homogenized in an ice bath. After centrifugation at 4000×*g* for 10 min at 4 °C, the supernatant was collected. A microplate reader was preheated for 30 min, and the wavelength was adjusted to 520 nm; OD values were adjusted to zero with distilled water. The iron content in intestinal tissue was detected using a tissue iron content detection kit (Beijing Solarbio Science & Technology Co., Ltd., catalog number: BC4355).

#### Analysis of the reduced GSH content in intestinal tissue and serum

(1) Frozen intestinal tissue (0.1 g) was weighed and fully ground using an automatic sample rapid grinder (Shanghai Jingxin, China). Then, the sample was centrifuged at 8000 rpm for 10 min at 4 °C; the supernatant was placed at 4 °C for subsequent analysis. (2) Anticoagulated blood was centrifuged at 4 °C at 600×*g* for 10 min. The upper layer (plasma) was transferred to another test tube and centrifuged at 4 °C at 8000×*g* for 10 min. The supernatant was transferred to a new test tube and placed at 4 °C for subsequent analysis. GSH in intestinal tissue and serum was detected using a reduced GSH content assay kit (catalog no.: BC1175, Beijing Solarbio Science & Technology Co., Ltd.).

#### Analysis of GPX4 activity

Approximately 0.1 g of rat intestinal tissue stored at − 80 °C was fully ground using an automatic sample rapid grinder (Shanghai Jingxin, China). After centrifugation, the supernatant was collected. The corresponding reagents were added in accordance with the manual provided with the intestinal GPX4 detection kit (Catalog No.:K003083P Beijing Solarbio Science & Technology Co., Ltd.). Then, the corresponding OD values were obtained using a microplate reader at a wavelength of 450 nm, and the results were analyzed.

#### Analysis of MDA content

The peroxidation product MDA combines with TBA to form a red product, with a maximum absorption peak at a wavelength of 532 nm. The OD value of the product is measured using a microplate reader, and then, the MDA content is calculated. The degree of lipid oxidation can be assessed by determining the MDA concentration. Briefly, approximately 0.1 g of intestinal tissue was collected, homogenized, and centrifuged at 8000*g* at 4 °C for 10 min. The supernatant was collected and placed on ice for testing. After anticoagulated blood was centrifuged, the upper layer (serum) was collected to measure the MDA content in intestinal tissue and serum using an MDA content assay kit (Catalog No.: BC0025, Beijing Solarbio Science & Technology Co., Ltd.).

#### Determination of IL-6 level of serum inflammatory cytokines in rats

Rat IL-6 ELISA KIT (item number: SEKR-0005, Beijing Solebo Biotechnology Co., Ltd.) was used to detect the level of serum inflammatory cytokine IL-6 in rats.

#### Determination of the activity of intestinal mucosal injury markers

Diamine oxidase (DAO) is widely found in animals (intestinal mucosa, lung, liver, kidney, etc.), plants and microorganisms. The activity of catalytic oxidation of polyamines to aldehydes is closely related to the synthesis of proteins and can reflect the integrity and damage of intestinal tissue. The activity of 500 nm was calculated by measuring the absorbance at the wavelength of DAO. DAO activity detection kit (item number: BC1280, Beijing Solebao Biotechnology Co., Ltd.) was used to detect DAO activity in intestinal tissue.

#### Determination of superoxide dismutase activity

Superoxide dismutase (SOD) is the main enzyme of antioxidation in the body, which can protect the cells from oxidative damage by scavenging ·O2^–^. Strictly follow the SOD activity test kit (item number: BC0170, Beijing Solebao Biotechnology Co., Ltd.) for testing.

### Determination of the expression of xCT and GPX4 in rat intestine

The expression of GPX4 and xCT was analyzed by Western blot. Ileal tissue (0.1 g) was collected, homogenized, and centrifuged at 13,400*g* at 4 °C for 5 min, and the supernatant was collected. The protein concentration was quantified using the BCA method. After the samples were boiled and denatured, the protein was separated using 10% sodium dodecyl sulfate–polyacrylamide gel electrophoresis (SDS‒PAGE) and then transferred to membranes, which were then blocked with 5% skimmed milk powder at room temperature for 2 h. Primary antibodies (anti-ferritin heavy chain (dilution 1:1000, Abcam, UK), anti-glutathione peroxidase 4 (dilution 1:1000, Abcam, UK), anti-xCT (dilution 1:1000, Abcam, UK), and anti-β-actin (dilution 1:5000, Abcam, UK)) were added separately, and the membranes were incubated at 4 °C overnight. Anti-rabbit secondary antibodies (dilution 1:5000, Beijing Zhongshan Golden Bridge Biotechnology Co., Ltd.) were added, and the membranes were incubated for 30 min at room temperature. Chemiluminescence development and fixation was performed. An AlphaEaseFC software processing system was used for the analysis, and the ratio of the gray value of the target protein band to the gray value of the β-actin band was used to reflect the expression level of the target protein.

### Statistical analysis

SPSS 26.0 was used to establish a database and perform statistical analyses. Data conforming to a normal distribution are expressed as the mean and standard deviation ($\overline{x}$ ± *s*). Repeated measurement analysis of variance was used to compare the differences among multiple groups, Then compare the differences between the two groups, when the variance is uneven, use Dunnett'st test; when the variance is uniform, use LSD-t test. *P* < 0.05 was considered statistically significant.

## Results

### Chiu’s scores for small intestinal tissue from rats in each group; pathological changes in the small intestine of rats in each group (Figs. 1, 2)

**Figure 1:**
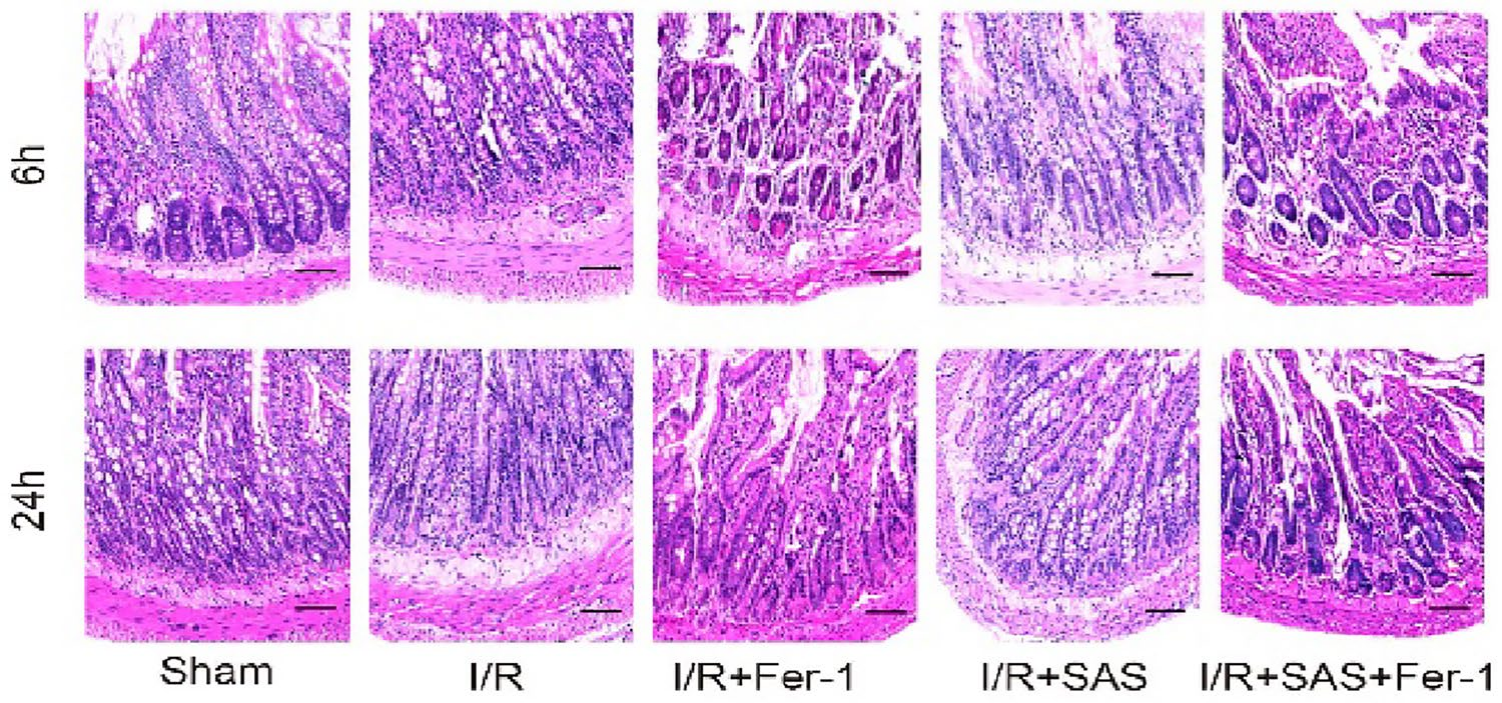
Hematoxylin–eosin (HE, ×200) staining of intestine tissue; scale bar is 100 μm.

**Figure 2:**
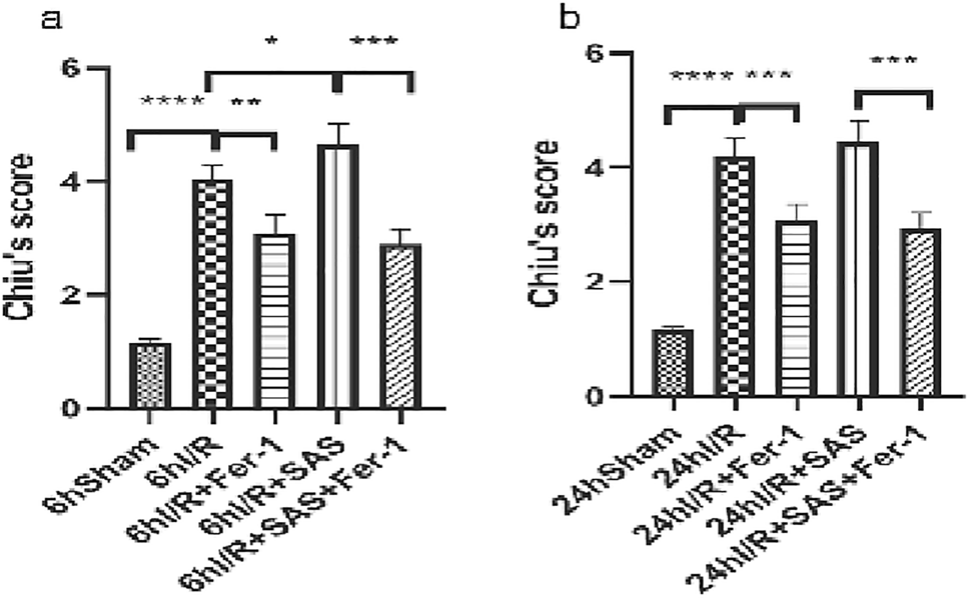
(**a,b**) Chiu’s pathology scores at 6 and 24 h after reperfusion, representing intestinal injury. The results are expressed as the mean ± S.D. *n* = 6. *p < 0.05, **p < 0.01, ***p < 0.001, ****p < 0.0001 by *t* tests.

The mucosal structure of the small intestine in the sham group was basically normal, with intact and orderly villi under 200× light microscopy. In the I/R group, the epithelial cells of the small intestinal mucosa were necrotic and detached; some of the villi were detached, and the lamina propria was exposed; and ulcers and hemorrhagic foci were visible. In the I/R + Fer-1 group, the damage to the small intestinal mucosal villi was milder than that in the I/R group, with mild mucosal edema and no obvious ulcers or hemorrhagic foci. In the I/R + SAS group, small intestinal mucosal villi injury was more severe than that in the I/R group; there were detached small intestinal villi, scattered ulcers and hemorrhagic foci. The degree of small intestinal mucosal villus injury in the I/R + SAS + Fer-1 group was improved compared with that in the SAS group.

### The ratio of W/D in rat intestine

**Figure 3:**
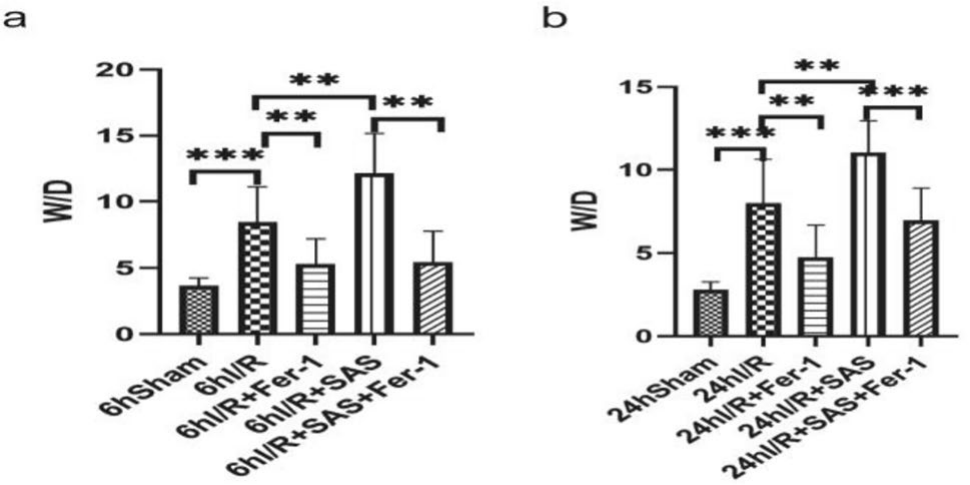
The changes of intestinal W/D ratio in each group after liver cold I/R. Intestinal sections were taken at 6 h and 24 h after reperfusion. The data are shown as the means ± SD (n = 6 per group). *p < 0.05, **p < 0.01, ***p < 0.001, ****p < 0.0001 by *t* tests.

At 6 and 24 h after modeling, the ratio of W/D in the other four experimental groups was higher than that in the Sham group(*P* < 0.05); the ratio of W/D in the inhibitor Fer-1 group was lower than that in the I/R group(*P* < 0.05), The ratio of W/D increased in I/R + SAS group(*P* < 0.05); compared with I/R + SAS group, the ratio of W/D in I/R + SAS + Fer-1 group decreased(*P* < 0.05) (Fig. 3).

### Serum IL-6, MDA levels (Fig. 4); intestinal MDA, SOD, DAO and intestinal Fe^2+^, GSH, GPX4 levels (Figs. 5, 6)

**Figure 4:**
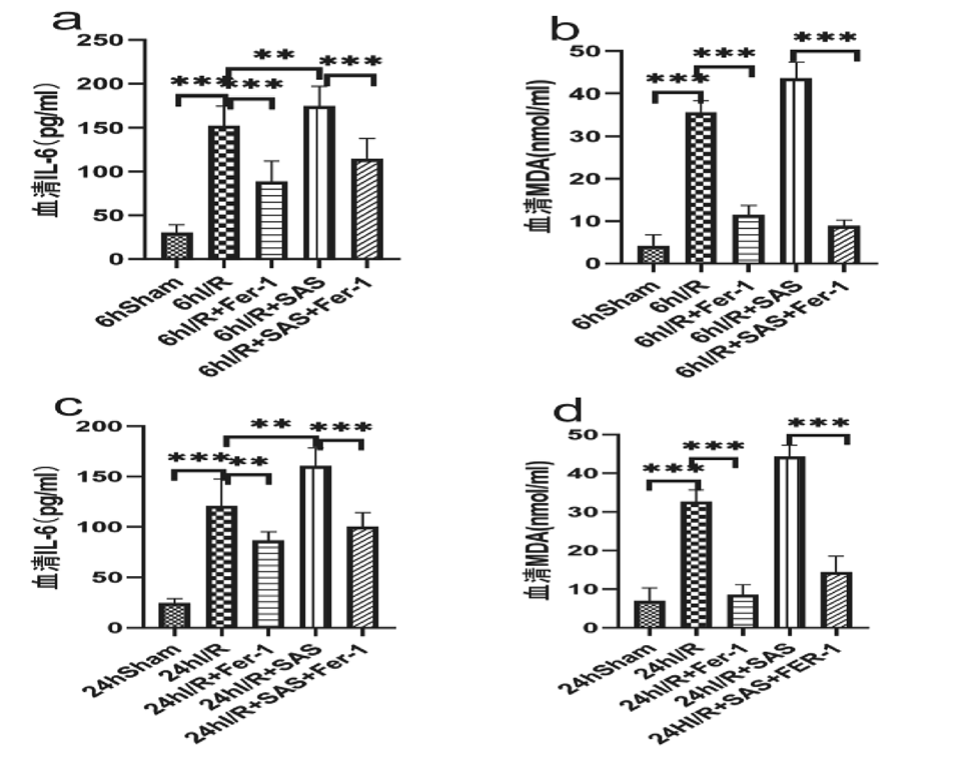
(**a,c**) The total IL-6,MDA levels in the serum at 6 h of reperfusion. (b,d) The total IL-6,MDA levels in the serum at 24 h of reperfusion. The results are expressed as the mean ± SD. *n* = 6. *p < 0.05, **p < 0.01, ***p < 0.001, ****p < 0.0001 by *t* tests.

**Figure 5:**
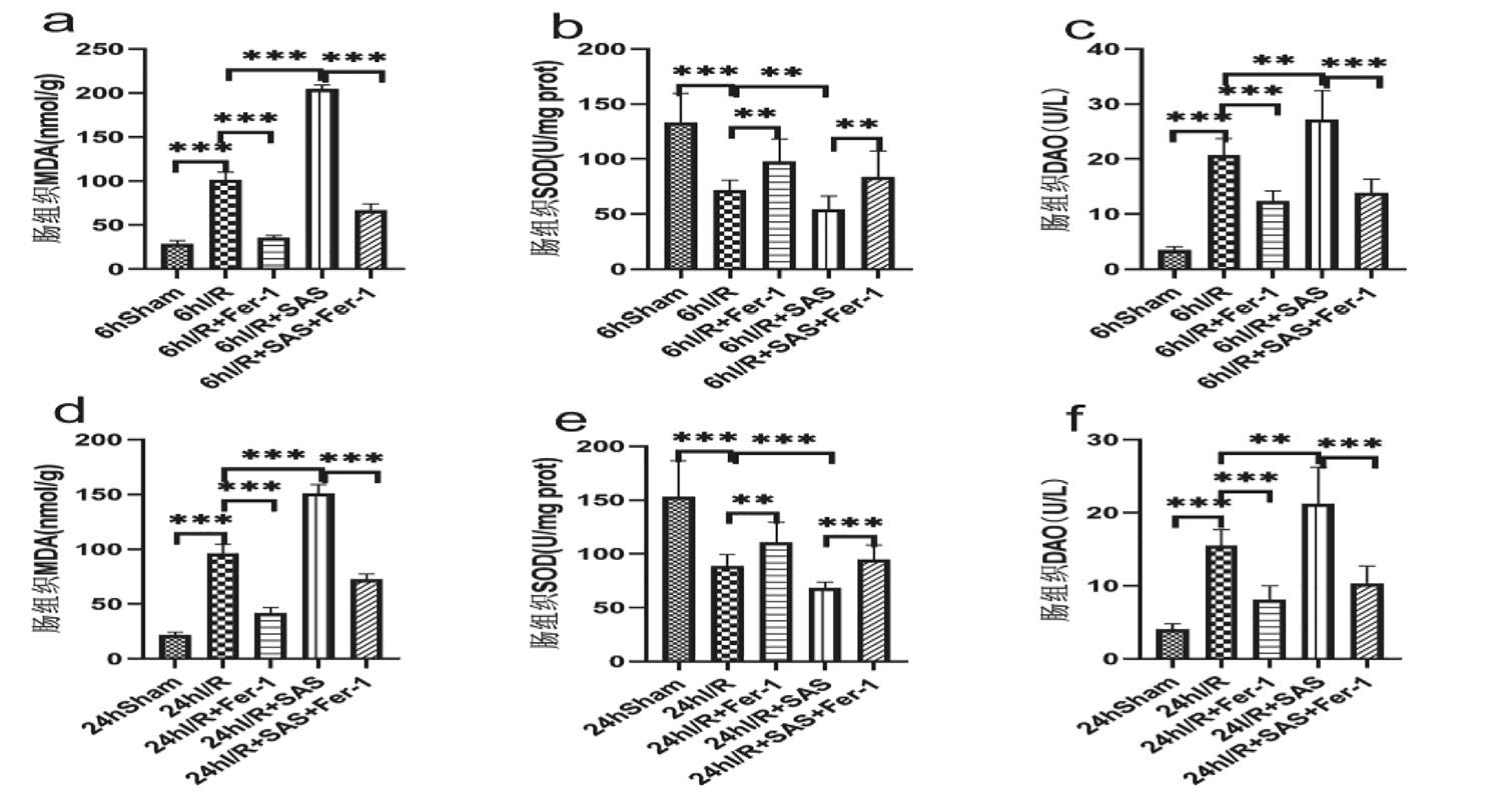
(**a–c**) The contents of MDA, SOD and DAO in liver tissue of rats in each group were measured 6 h after liver I/R. (d–f) The changes of MDA, SOD and DAO in liver tissue were observed 24 h after I/R. The data are shown as the means ± S D (*n* = 6 per group). *p < 0.05, **p < 0.01, ***p < 0.001, ****p < 0.0001 by *t* tests.

**Figure 6:**
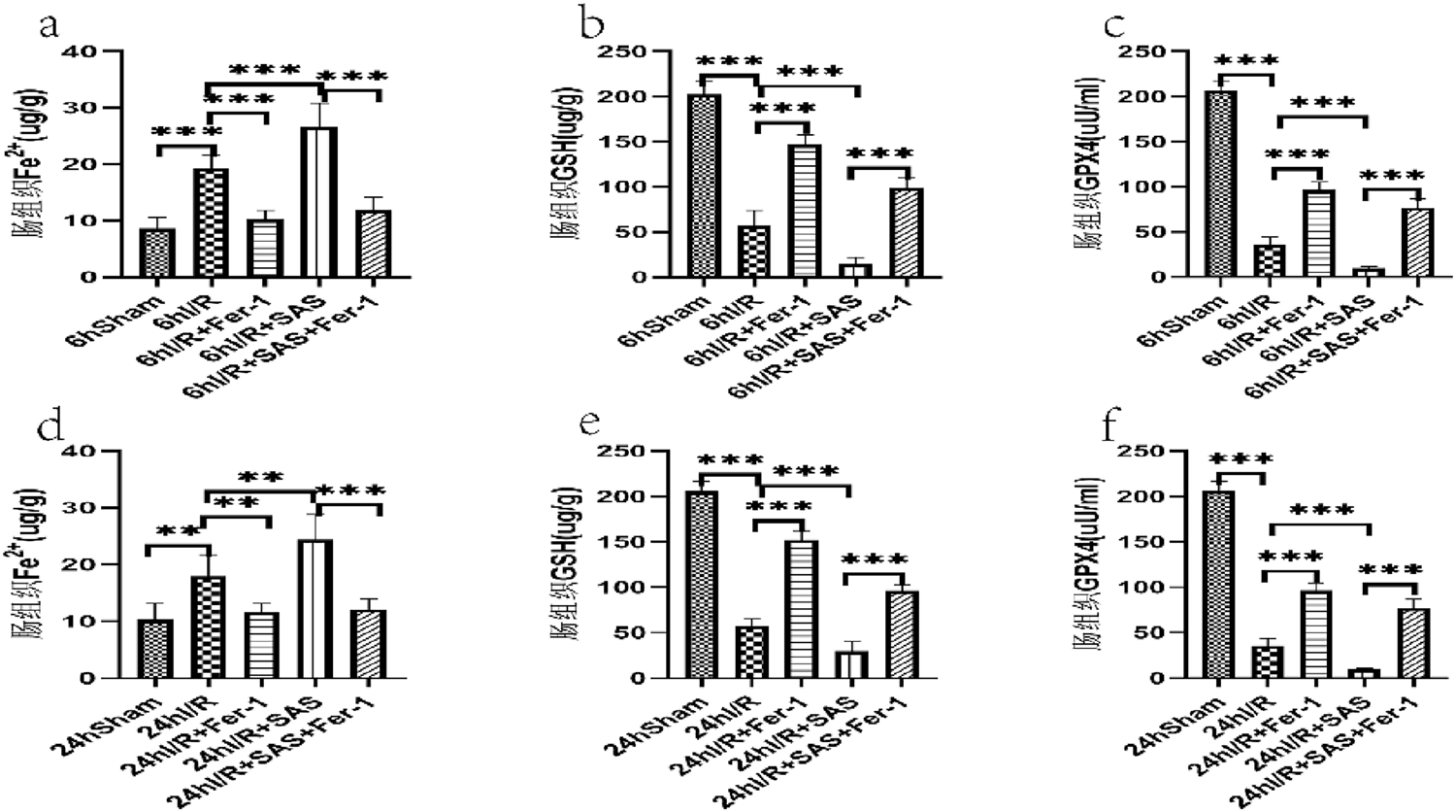
(**a–c**)The levels of Fe^2+^, GSH, GPX4 at 6 h after liver I/R in each group. (**d–f**) The levels of Fe^2+^, GSH, GPX4 at 24 h f after liver I/R in each group. The data are shown as the means ± S D (*n* = 6 per group). *p < 0.05, **p < 0.01, ***p < 0.001, ****p < 0.0001 by *t* tests.

The blood of inferior hepatic vena cava of each group was taken at 6 and 24 h after modeling. After centrifugation, the upper serum was taken to detect serum IL-6 and evaluate inflammation; Serum MDA was detected to evaluate the level of oxidative stress. Compared with the Sham group, the contents of serum IL-6 and MDA in the intestinal tissue of the other four groups increased significantly at each time point after reperfusion (*P* < 0.05); compared with I/R, the contents of serum IL-6 and MDA decreased in I/R + Fer-1 group at 6 h and 24 h after intestinal reperfusion (*P* < 0.05), The contents of serum IL-6 and MDA increased 6 h and 24 h after intestinal reperfusion in I/R + SAS group (*P* < 0.05); compared with I/R + SAS group, the contents of serum IL-6 and MDA decreased in I/R + SAS + Fer-1 group at 6 h and 24 h after intestinal reperfusion (*P* < 0.05).

After 6 and 24 h of intestinal injury, the ileal homogenate of rats in each group was taken, and the supernatant was centrifuged to detect the contents of MDA and SOD in intestinal tissue in order to evaluate the level of oxidative stress in intestinal tissue; Detection of DAO in intestinal tissue to evaluate intestinal injury. Compared with Sham group, the contents of MDA and DAO in intestinal tissue of other experimental groups increased significantly at each time point, while the activity of SOD in intestinal homogenate decreased (*P* < 0.05); Compared with the model group, I/R + Fer-1 group significantly increased the activity of SOD and decreased the content of MDA and DAO in intestinal tissue of rats at each time point after intestinal injury (*P* < 0.05), I/R + SAS group significantly decreased the activity of SOD and increased the content of MDA and DAO in intestinal tissue of rats at each time point after intestinal injury (*P* < 0.05); compared with I/R + SAS, I/R + SAS + Fer-1 group significantly increased the activity of SOD and decreased the levels of MDA and DAO in intestinal tissue at each time point after intestinal injury in rats (*P* < 0.05).

Compared with the Sham group at 6 h after reperfusion, the intestinal tissue GSH and GPX4 decreased and the Fe^2+^ content increased in the I/R group(*P* < 0.05); compared with I/R group at 6 h after reperfusion, the contents of MDA and Fe^2+^ in intestinal tissue of I/R + Fer-1 group decreased, while GSH and GPX4 in intestinal tissue increased (*P* < 0.05); compared with I/R group at 6 h after reperfusion, intestinal Fe^2+^ content increased and intestinal tissue GSH and GPX4 decreased in I/R + SAS group (*P* < 0.05); compared with I/R + SAS group at 6 h after reperfusion, Fe^2+^ content decreased and intestinal GSH and GPX4 increased in I/R + SAS + Fer-1 group (*P* < 0.05). Compared with the five groups after 6 h of reperfusion, the change trend of each index of the five groups after 24 h of reperfusion was basically the same. But in I/R + Fer-1 group, the decrease of Fe^2+^ in intestinal tissue at 6 h after reperfusion was more significant than that at 24 h after reperfusion; In I/R + SAS group, intestinal GSH decreased more significantly at 6 h after reperfusion than that at 24 h after reperfusion, and the content of Fe^2+^ in intestinal tissue increased more significantly at 6 h after reperfusion. In I/R + SAS + Fer-1 group, the content of Fe^2+^ in intestinal tissue of 6 h reperfusion group was significantly lower than that of 24 h reperfusion group.

### Expression of GPX4 and xCT in ileal tissue of rats in each group (Fig. 7)

**Figure 7:**
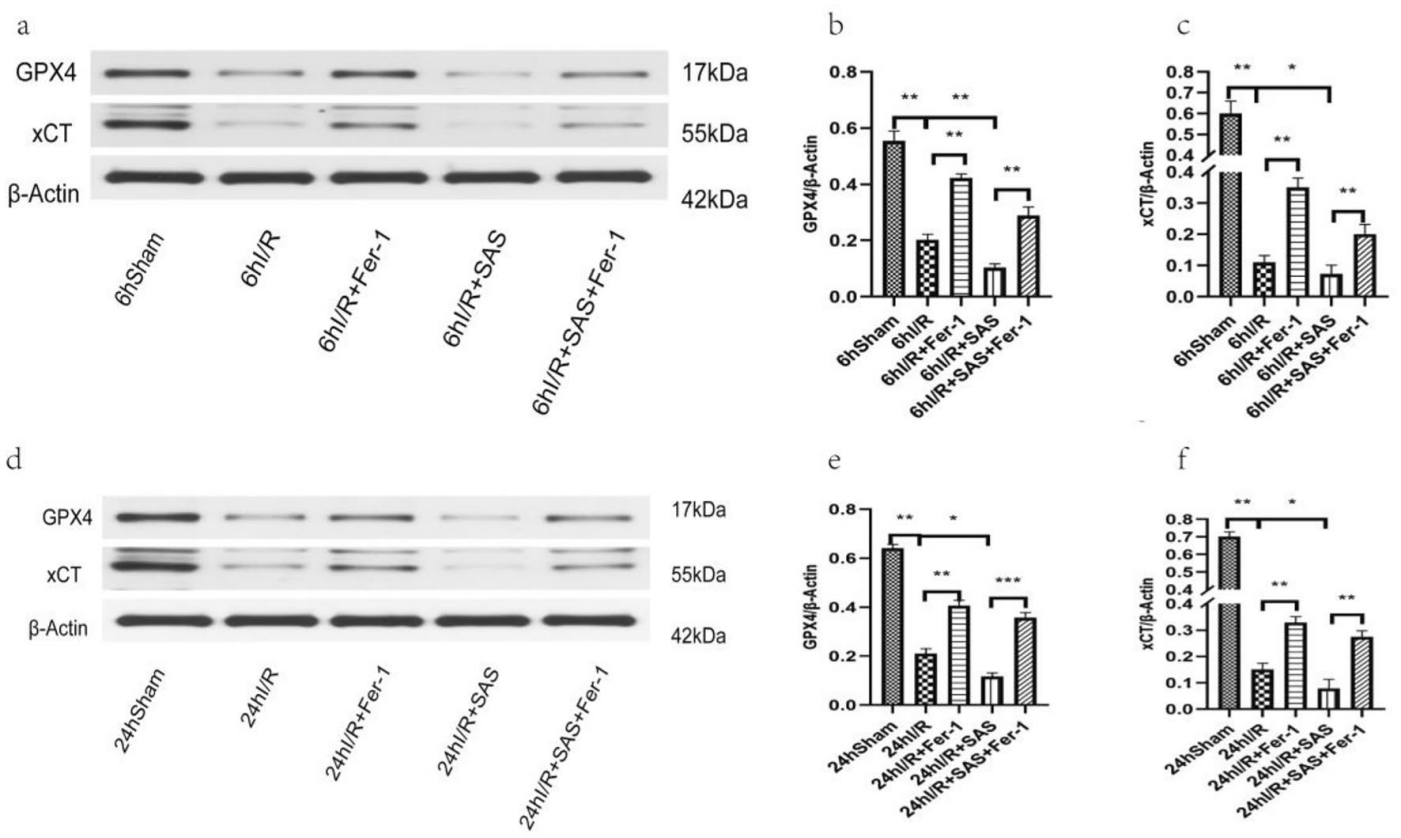
Ileal tissues were collected from SD rats 6 h and 24 h after ischemia–reperfusion. The expression of GPX4 and xCT was determined by Western blot method (*n* = six). (**a–c**) The Western blot results of GPX4 and xCT for each group at 6 h after reperfusion; (**d–f**) the Western blot results of GPX4 and xCT for each group 24 h after reperfusion. There was a significant difference in the levels of GPX4 and xCT expression in the ileal tissues of the five groups at both time points. All results are expressed as the mean ± S.D. *n* = six. *p < 0.05, **p < 0.01, ***p < 0.001, ****p < 0.0001 by *t* tests.

Compared with those in the Sham group, the expression levels of GPX4 and xCT in the I/R group were lower (*P* < 0.05); compared with those in the I/R group, the expression levels of GPX4 and xCT in the Fer-1 group were higher (*P* < 0.05); compared with those in the I/R group, the expression levels of GPX4 and xCT in the SAS group were lower (*P* < 0.05); and compared with those in the I/R + SAS group, the expression levels of GPX4 and xCT in the I/R + SAS + FER-1 group were higher (*P* < 0.05). In addition, in the process of AOLT liver I/R in rats, the trends for changes in all indicators was basically the same at 6 h and 24 h after reperfusion, and the differences were not significant.

## Discussion

In this study, the intestinal injury model of autologous orthotopic liver transplantation in SD rats was established with reference to literature^12^. The anhepatic phase was 30 ± 1 min, and data were collected for 2 time points after reperfusion: 6 h and 24 h. The results showed that compared with that of rats in the sham group, the intestinal mucosal structure of the rats in the I/R group was damaged; Chiu's score, W/D, serum MDA, serum IL-6, and intestinal MDA, DAO, Fe^2+^ levels were higher; and intestinal SOD, GSH and GPX4 levels were lower. These results suggest that the model was established successfully.

The close connection between the intestine and the liver is referred to as the intestine-liver axis. Liver dysfunction is a very important factor leading to the occurrence of liver-related diseases. Additionally, maintaining the relative stability of the internal environment of the body requires sound intestinal homeostasis and good hepatoprotective mechanisms^15^. In the perioperative period of LT, the homeostasis of the intestine-liver axis is disrupted due to I/R, causing a series of pathophysiological changes.

Ferroptosis is a new form of regulatory programmed cell death, and its occurrence is associated with the excessive accumulation of L-ROS^16,17^. Ferroptosis is substantially different from necrosis, apoptosis and other forms of cell death in terms of cell morphology, gene regulation, and metabolism. This unique mode of cell death, which is caused by iron-dependent lipid peroxidation, is regulated by a variety of metabolic pathways, including iron ion levels, amino acid metabolism, lipid metabolism, mitochondrial activity, redox homeostasis, and various disease-related signaling pathways^18–21^. Studies have found that ferroptotic cells trigger the innate immune system by releasing inflammation-related injury-related molecules and that immune cells trigger inflammatory responses by recognizing different modes of cell death mechanisms^22^. Studies have also shown that ferroptosis occurs during I/R injury and aggravates I/R injury^8,9^. I/R injury is the main issue with LT. I/R injury in LT can cause damage to the liver itself and may also lead to damage to the body’s distant organs.

Ferrostain-1 is an effective and selective ferroptosis inhibitor. It prevents membrane lipid damage through a reduction mechanism and eliminates lipid ROS produced by the body by inhibiting the enzyme-mediated process catalyzed by iron-dependent lipoxygenase, thereby inhibiting cell death^23–28^. SAS is an important drug for the treatment of ulcerative colitis and is also one of the most commonly used anti-inflammatory drugs in clinical practice. SAS also plays an important role in the treatment of arthritis and retinal inflammatory lesions. Studies have shown that in the treatment of human fibrosarcoma, SAS can inhibit the expression of system Xc on the fibrosarcoma cell membrane, suggesting that SAS can inhibit the occurrence and development of tumors to a certain extent. The possible mechanism by which SAS induces ferroptosis is by interrupting the cystine uptake signaling pathway^29–38^. System Xc¯ is one of the key signal pathways of ferroptosis. The functional subunit xCT of system Xc¯ is a system Xc¯ light chain protein encoded by the SLC7A11 gene.^.^ xCT regulates the exchange of intracellular glutamate with extracellular cystine. Therefore, the expression level of system Xc¯ can be reflected by detecting the expression level of xCT^39^.

Selection of drug dose in this study reference^40–42^ and pre-experimental results. In the pre-experiment, three doses of Ferrostain-1 were selected and divided into low, middle and high dose groups, which were intraperitoneally injected with 1.0, 5.0 and 10.0 mg/kg 1 hour before modeling. Compared with the middle dose group, the intestinal tissue and serum detection index levels of the middle dose group were improved more significantly, and the results of the low dose group did not meet the expectations; both the high dose group and the middle dose group could achieve the expected results, but the improvement of each index in the high dose group was not more significant than that in the middle dose group. Therefore, combined with the previous pre-experimental results, Ferrostain-1 5.0 mg/kg was injected intraperitoneally 1 h before modeling in this study. Three doses of SAS were used in the pre-experiment, which were divided into low, middle and high dose groups, which were injected intraperitoneally with 100, 250 and 500 mg/kg, respectively. There was no significant difference in the levels of intestinal tissue and serum between the low dose group and the I/R group; the results of some test indexes in the middle dose group can not meet the expectations; the detection results of the high dose group could reach the expectation, and there was significant difference between the high dose group and the I/R group. Therefore, combined with the results of previous animal experiments, this experiment chose continuous intraperitoneal injection of SAS 500 mg/kg before making membrane for 7 days.

The results of this study showed that ferroptosis occurred in rats after autologous LT liver cold I/R-induced intestinal injury and that the long-term high-dose intraperitoneal injection of SAS further increased the risk of ferroptosis in rats after AOLT intestinal injury. The intraperitoneal injection of ferrostatin-1 1 h before AOLT surgery in rats improved intestinal injury in rats after I/R, at the same time reduce the release of inflammatory cytokines IL-6 and had a certain protective effect on the intestinal tract of rats. SAS may induce ferroptosis in intestinal tissue by suppressing system Xc¯ channels and regulating the exchange of intracellular glutamate with extracellular cystine. In the I/R group and the SAS group, the Fe^2+^ content in intestinal tissue was high, and iron overload was observed; the increase in the SAS group was more significant than that in the I/R group. It can be speculated that ferroptosis caused by LT-induced intestinal injury in rats may also be related to iron overload. Notably, this study has limitations. What is the occurrence stage and gene regulation target of ferroptosis in intestinal injury caused after I/R during the perioperative period of liver transplantation. In addition, whether it can be achieved by inhibiting ferroptosis to achieve clinical targeted treatment of intestinal injury induced by I/R in liver transplantation is not clear. But these issues were not the focus of our study and do not affect the reliability of the conclusions; however, they will be explored in future studies (Supplementary Information).

## Conclusion

Ferroptosis is involved in the pathophysiological process of AOLT liver cold I/R-induced intestinal injury in rats. Additionally, SAS may increase the risk of rat AOLT liver cold I/R-induced intestinal injury by inhibiting the System Xc¯/GSH/GPX4 pathway or driving iron overload after I/R.

### Supplementary Information

Supplementary Information.

## Data Availability

All data generated or analysed during this study are included in this article, the datasets used during the current study available from the corresponding author on reasonable request.

## Abbreviations

AOLT: Autogenous orthotopic liver transplantation
I/R: Ischemia reperfusion
L-ROS: Lipid reactive oxygen species
FER-1: Ferrostatin-1
SAS: Sulfasalazine
SPF: Specific pathogen free
H: Hour
DMSO: Dimethyl sulfoxide
MDA: Malondialdehyde
PV: Portal vein
IL-6: Interleukin-6
ELISA: Enzyme-linked immunosorbent assay
SOD: Superoxide dismutase
GSH: Glutathione
GPX4: Glutathione peroxidase 4
System Xc¯: Cystine/glutamate antiporters
LT: Liver transplantation
SHVC: Suprahepatic vena cava
IHVC: Infrahepatic vena cava
BD: Evacuated blood collection
H&E: Hematoxin Eosin
W/D: Wet/dry
OD: Optical density
Rpm: Revolutions per minute
TBA: Thiobarbituric acid
DAO: Diamine oxidase
