# Supplementary material for: Effect of sulfasalazine on ferroptosis during intestinal injury in rats after liver transplantation
Source: Sci Rep. 2024 Mar 28;14:7349. doi: 10.1038/s41598-024-58057-z (PMC10973495; doi:10.1038/s41598-024-58057-z)

**6h original blots in supplementary information and cropped blots in main paper：**


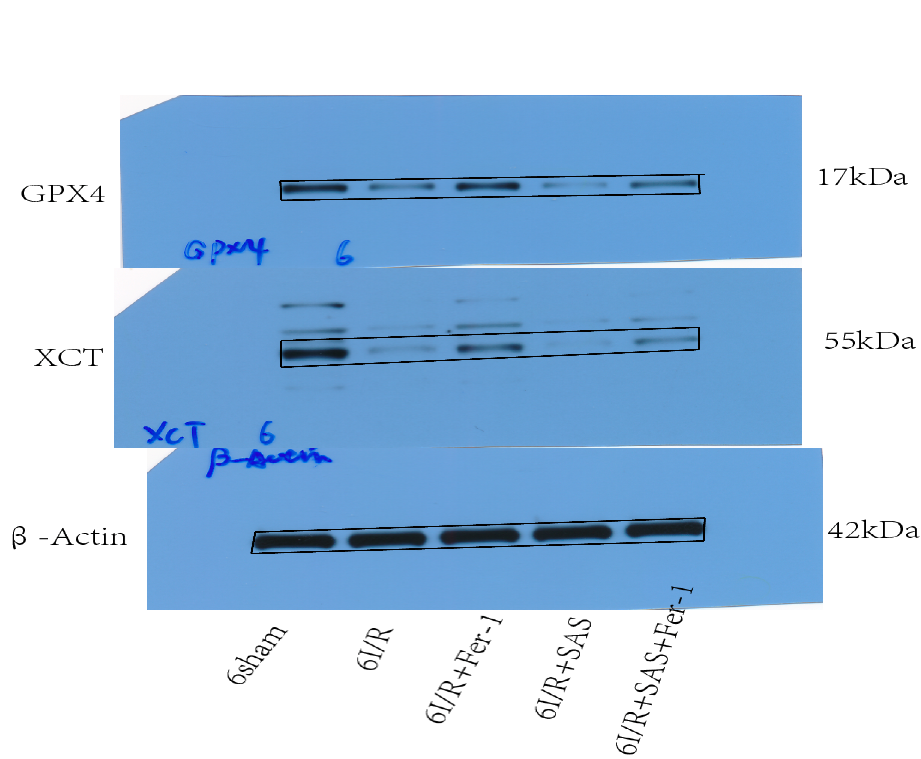


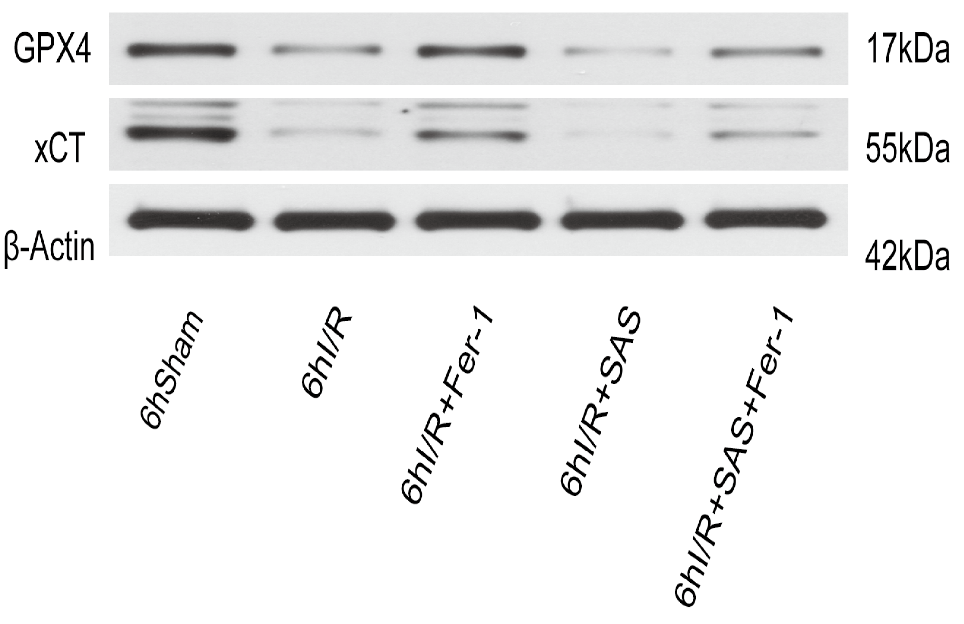


**24h original blots in supplementary information and cropped blots in main paper：**


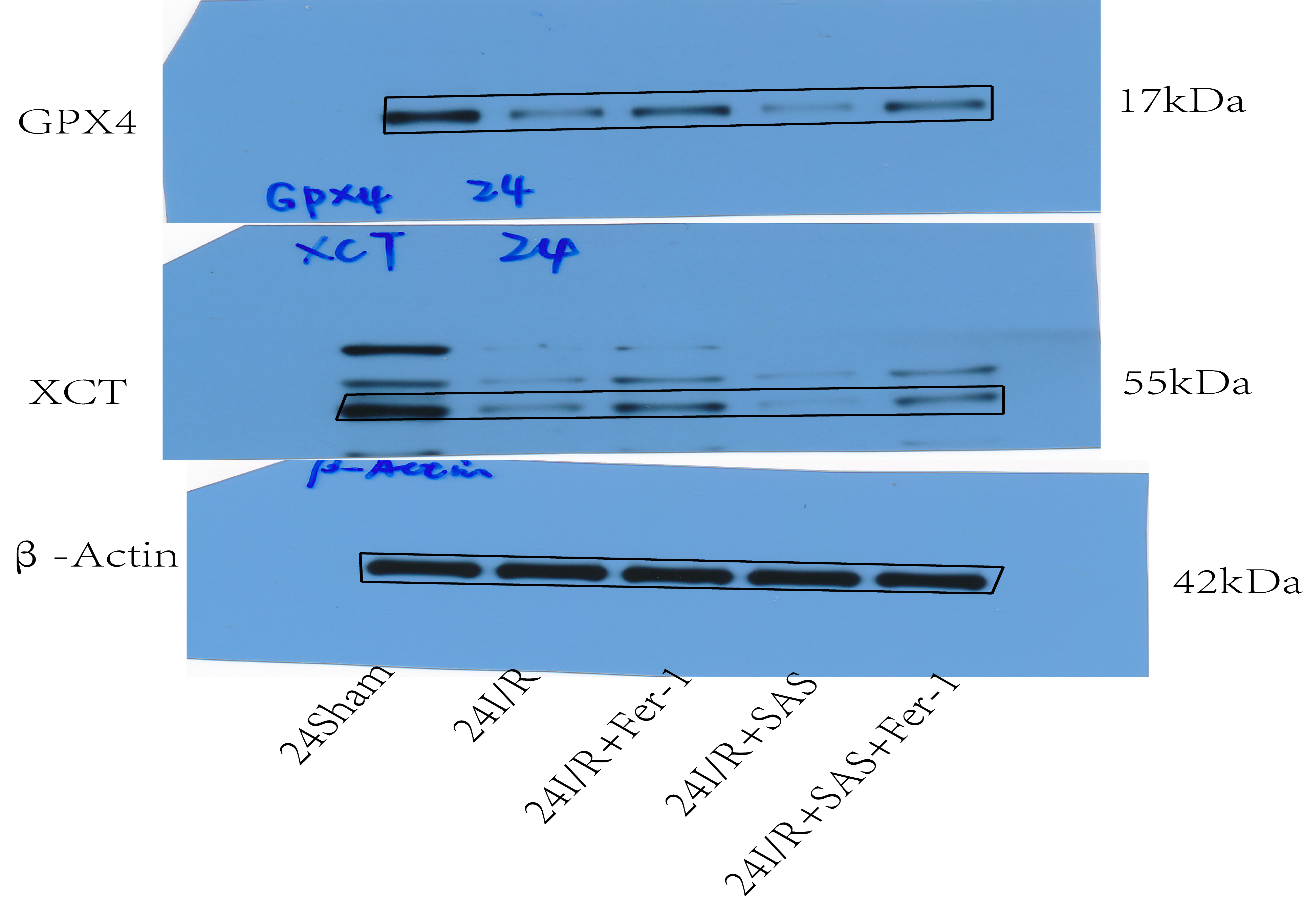


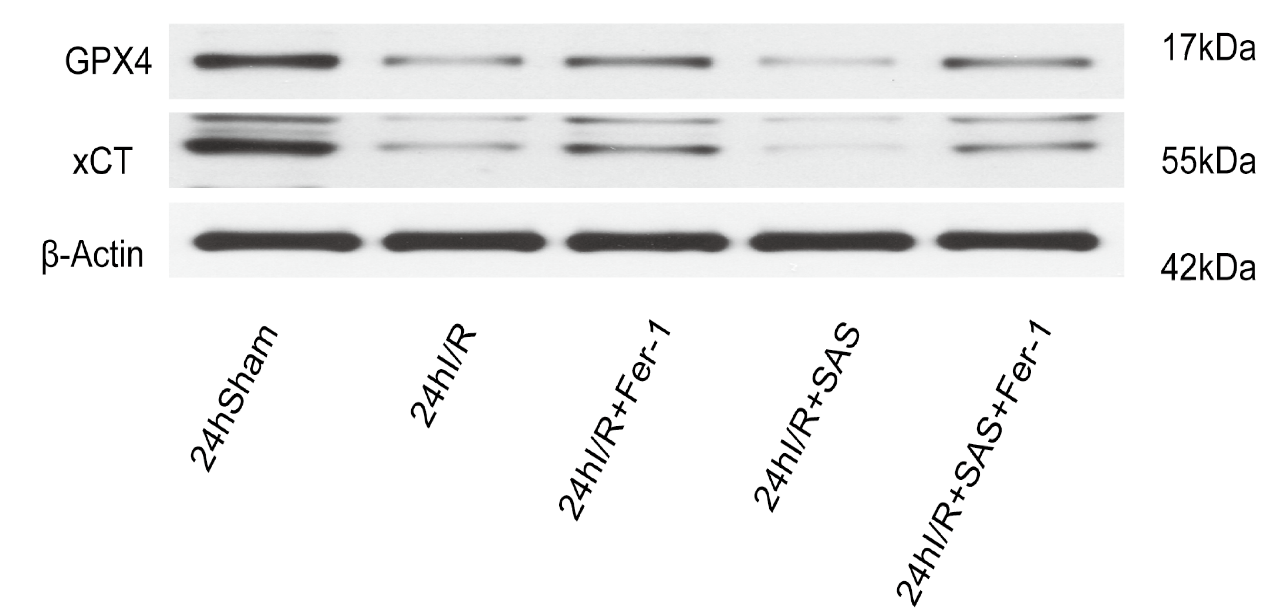

Supplement: Supplementary file 1 — Supplementary Information. [file 41598_2024_58057_MOESM1_ESM.docx]
